# Integrated Studies on the Biological Functions and Regulatory Mechanisms of Dedicator of Cytokinesis Family Proteins—Secondary Publication

**DOI:** 10.31662/jmaj.2025-0460

**Published:** 2026-08-21

**Authors:** Yoshinori Fukui

**Affiliations:** 1 Medical Institute of Bioregulation, Kyushu University, Fukuoka, Japan

**Keywords:** small GTPases, DOCK family proteins, cell motility, immunological synapse, atopic dermatitis

## Abstract

Small guanosine triphosphatases act as molecular switches in signaling pathways by alternating between an inactive guanosine diphosphate (GDP)–bound state and an active guanosine triphosphate (GTP)–bound state. Upon activation, they interact with various effector molecules to regulate a wide range of cellular processes. This activation is triggered by external stimuli and is mediated by guanine nucleotide exchange factors (GEFs). GEFs are classified into two major types: Dbl homology (DH)–domain-containing proteins (DH-type GEFs) and dedicator of cytokinesis (DOCK) proteins (DOCK-type GEFs). Traditionally, DH-type GEFs were thought to represent the universal GEFs in eukaryotic systems. However, since we first demonstrated the crucial role of DOCK2 in lymphocyte migration, accumulating evidence has highlighted the importance of DOCK family proteins in diverse biological responses. These proteins share two evolutionarily conserved domains: DOCK homology region-1 (DHR-1) and DHR-2. The DHR-1 domain mediates phospholipid binding, which governs the intracellular localization of DOCK proteins, whereas the DHR-2 domain functions as the catalytic site for GDP–GTP exchange reaction on Rac or Cdc42. In mammals, the DOCK family comprises 11 members. In this review, I summarize the biological functions and regulatory mechanisms of DOCK proteins, with a particular emphasis on DOCK2 and DOCK8.

## Structure and Expression of Dedicator of Cytokinesis 2 (DOCK2)

DOCK2 is a Rac-specific guanine nucleotide exchange factor (GEF) functioning downstream of various receptors, and its expression is largely restricted to hematopoietic cells ^(1), (2)^. Like other DOCK-type GEFs, DOCK2 possess two evolutionarily conserved domains: DOCK homology region-1 (DHR-1) and DHR-2 ^(3)^. The DHR-1 domain regulates the intracellular localization of DOCK2 through the interaction with PtdIns(3,4,5)P_3_^(4)^. In contrast, the DHR-2 domain interacts with Rac and catalyzes the guanosine diphosphate–guanosine triphosphate exchange reaction on Rac. Among the amino acid residues encoding DOCK2 DHR-2 domain, the valine residue at position 1,538 plays a key role by functioning as a nucleotide sensor, and its mutation to alanine completely abrogates the Rac GEF activity of DOCK2 ^(2), (5)^.

## Lymphocyte Migration and DOCK2

After completing their development in the thymus and bone marrow, T and B lymphocytes enter the bloodstream and migrate into the secondary lymphoid organs—such as the lymph nodes, spleen, and Peyer’s patches—where they encounter foreign antigens collected from peripheral tissues and initiate antigen-specific immune responses. Although this migratory response is driven by chemokines, the specific Rac GEFs acting downstream of chemokine receptors had remained unidentified. To clarify the role of DOCK2 in lymphocyte migration, we generated DOCK2-deficient (*Dock2^–/–^*) mice ^(1)^. When T and B lymphocytes from control (*Dock2^+/–^*) mice were stimulated in vitro with chemokines such as CCL21 and CXCL13, they exhibited robust, dose-dependent migration. However, DOCK2-deficient lymphocytes did not show any migratory responses to these chemokines, resulting in profound atrophy of the secondary lymphoid organs ^(1), (6)^.

Chemokine receptors signal through heterotrimeric G proteins ^(1)^. Upon chemokine binding, these G proteins dissociate into an α-subunit and βγ-subunits, the latter activating multiple downstream signaling pathways. For example, chemokine stimulation induced Akt phosphorylation and Ca^2+^ mobilization normally even in the case of DOCK2-deficient lymphocytes ^(1)^. However, Rac activation and actin polymerization triggered by chemokines were severely impaired in the absence of DOCK2 ^(1)^. Consistent with these findings, two-photon microscopy revealed that DOCK2 deficiency markedly reduced the motility of T and B cells within the lymphoid organs of live mice ^(7)^. Together, these results establish DOCK2 as a Rac GEF that is indispensable for lymphocyte migration and homing (Figure 1).

**Figure 1.**
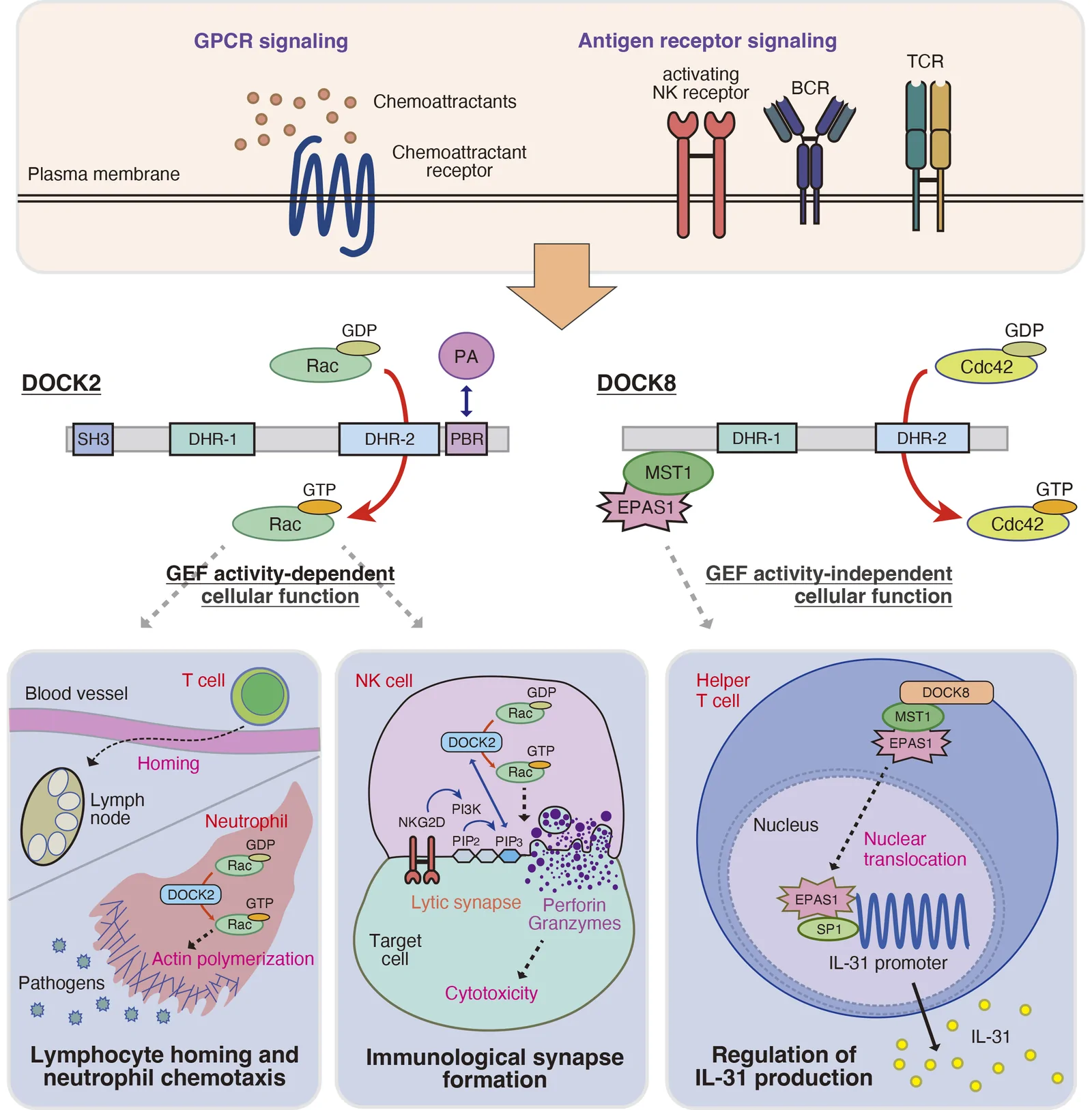
Leukocyte functions regulated by DOCK2 and DOCK8 through the GEF activity–dependent or –independent mechanisms. DOCK2 is a Rac-specific GEF and acts downstream of various receptors, including antigen receptors, chemokine receptors, and NK receptors. When the expression of DOCK2 is genetically deleted, Rac activation mediated by these receptors is almost completely abolished, and the associated cellular functions are severely impaired. Thus, DOCK2 regulates the migration and activation of leukocytes by functioning as the Rac GEF. In contrast, IL-31 is a major pruritogen associated with atopic dermatitis. We found that DOCK8 negatively regulates IL-31 production by associating with the transcription factor EPAS1 and inhibiting its nuclear translocation. Thus, in this context, DOCK8 regulates IL-31 production in helper T cells, independently of the Cdc42 GEF activity, through its adaptor functions. DOCK: dedicator of cytokinesis; GEF: guanine nucleotide exchange factor; IL: interleukin; NK: natural killer.

## Immunological Synapse Formation and DOCK2

Engagement of antigen receptors with antigens triggers the formation of an immunological synapse (IS) at the interface between lymphocytes and antigen-presenting cells (APCs) or target cells. Traditionally, Vav proteins, one of the Dbl homology-type GEFs expressed in lymphocytes, have been regarded as the primary Rac GEFs acting downstream of antigen receptors ^(8), (9), (10), (11), (12), (13), (14)^. However, we observed that antigen receptor–mediated Rac activation was severely impaired in DOCK2-deficient T cells, B cells, and natural killer (NK) cells ^(15), (16), (17)^. Importantly, it has been shown that immunological defects in Vav-deficient lymphocytes can be rescued by the expression of the Vav mutant lacking the Rac GEF activity ^(18)^, suggesting that Vav proteins primarily function as adaptor molecules rather than as essential Rac activators.

To investigate how DOCK2 deficiency impacts IS formation and T-cell functions, we employed transgenic mice expressing the 2B4 TCR, which recognizes the moth cytochrome C (MCC) peptide presented by I-E^k^ MHC class II molecules. When *Dock2^+/–^* CD4^+^ T cells expressing the 2B4 TCR were co-cultured with APCs in the presence of MCC peptide, TCR, LFA-1, PKC-θ, and lipid rafts readily accumulated at the T cell–APC interface. Antigen-induced translocation of PKC-θ and LFA-1 occurred normally even in *Dock2^–/–^* CD4^+^ T cells ^(15)^. Surprisingly, however, TCR polarization and lipid raft clustering were almost completely abolished in the absence of DOCK2 ^(15)^, leading to impaired T-cell proliferation. *Dock2^–/–^* 2B4 TCR transgenic mice also exhibited a marked reduction of double-positive thymocytes ^(15)^, suggesting that DOCK2 helps set the threshold for positive selection in the thymus. Additionally, we found that TCR stimulation–induced down-regulation of the interleukin (IL)-4Rα chain on the T-cell surface was impaired in the absence of DOCK2, resulting in sustained IL-4R signaling and excessive T helper type 2 responses ^(19)^. Thus, IS formation is involved in many checkpoints that affect “cell-fate decision” during the development and differentiation of lymphocytes.

The mechanisms controlling DOCK2-dependent IS formation were investigated using NK cells that function to protect the body against viral infection and tumor development through contact-dependent cytotoxicity. NK cells express various activating receptors, such as NKG2D, which recognizes the MHC class I–like ligand Rae1 on target cells. Engagement of the receptors with their ligands promotes receptor clustering at the cell–cell interface and initiates polarized trafficking of lytic granules toward the contact site. Although the defective IS formation observed in DOCK2-deficient NK cells was rescued by re-expressing wild-type DOCK2, the expression of the GEF-dead VA mutant failed to do so, indicating that DOCK2 controls lytic synapse formation through Rac activation (Figure 1) ^(17)^. In addition, we found that DOCK2 localizes to the synapse in a manner that depends on PI3K activation and PtdIns(3,4,5)P_3_ production (Figure 1) ^(17)^.

## Neutrophil Chemotaxis and DOCK2

Neutrophils are among the most intensively studied cells with respect to migration, where the coordinated action of PtdIns(3,4,5)P_3_ and Rac is crucial. In neutrophils, PtdIns(3,4,5)P_3_ is primarily generated by PI3Kγ; however, the identity of the Rac GEF involved had remained elusive. We found that DOCK2-deficient neutrophils exhibited markedly impaired activation of Rac1 and Rac2 in response to fMLP (a bacterial chemoattractant), resulting in reduced motility and loss of cell polarity ^(4)^. Furthermore, fMLP-induced production of reactive oxygen species (ROS) was significantly diminished to less than one-fifth of the wild-type level ^(4)^, confirming the role of DOCK2 as the critical Rac GEF for both neutrophil chemotaxis and ROS production (Figure 1).

To further investigate the mechanisms controlling intracellular DOCK2 dynamics, we generated knock-in mice expressing a DOCK2-GFP fusion protein ^(20)^. We discovered that, while PtdIns(3,4,5)P_3_ recruits DOCK2 to the plasma membrane, its further localization to the leading edge requires interaction with phosphatidic acid (PA) ^(20)^. Interestingly, the addition of PA, but not other acidic phospholipids, to wild-type neutrophils induced actin polymerization, a response absent in DOCK2-deficient cells ^(20)^. These results led us to propose a new model for leading edge formation during neutrophil chemotaxis, wherein the sequential generation of two phospholipids—PtdIns(3,4,5)P_3_ and PA—spatially and temporally regulates intracellular DOCK2 dynamics to focus Rac activation and stabilize the leading edge ^(20)^.

## Type I Interferon Production and DOCK2

Plasmacytoid dendritic cells (pDCs) produce not only inflammatory cytokines but also type I interferons (IFN-α and IFN-β) upon recognition of nucleic acid ligands by intracellular toll-like receptors 7 and 9 (TLR7/9). When DOCK2-deficient pDCs were stimulated with TLR7 ligand R848 and TLR9 ligand CpG-A, inflammatory cytokines such as IL-6 and IL-12p40 were normally produced; however, type I IFN production was severely impaired in the absence of DOCK2 ^(21)^. Similar results were obtained when a dominant negative Rac mutant was expressed in wild-type pDCs ^(21)^. Interestingly, CpG-A stimulation induced actin polymerization in wild-type pDCs, which was also observed in TLR9-deficient cells. In contrast, DOCK2-deficient pDCs failed to polymerize actin, correlating with a lack of Rac activation. IRF7, a key transcription factor for type I IFN gene expression, is regulated by IKKα. We found that IKKα activation was impaired in DOCK2-deficient pDCs ^(21)^. Collectively, these results unveil the novel regulatory mechanisms wherein the DOCK2–Rac–actin axis controls type I interferon induction, independently of TLR-mediated antigen recognition, through the activation of IKKα ^(21)^.

## Discovery of a DOCK2 Inhibitory Metabolite

As demonstrated thus far, DOCK2 is an indispensable Rac GEF that mediates immune cell migration and activation. As a result, DOCK2 deficiency in humans causes severe immunodeficiency ^(22)^. Surprisingly, however, we recently discovered cholesterol sulfate (CS) as an endogenous metabolite that can inhibit DOCK2 functions ^(23)^. CS binds to the DHR-2 domain of DOCK2 and inhibits its Rac GEF activity with an IC_50_ value of approximately 2 μM ^(23)^. This inhibitory effect was not observed with other cholesterol derivatives ^(23)^, indicating the specificity of CS. Consistent with these findings, treatment of T cells and neutrophils with CS dose-dependently suppressed chemotactic responses induced by CCL21 and fMLP, respectively ^(23)^. Interestingly, neutrophils chemotaxing in a Taxiscan chamber along the fMLP gradient abruptly stopped migration when CS was added to the same side as fMLP ^(23)^, raising the possibility that CS acts as a chemical barrier in vivo. CS is abundantly produced in mice in the Harderian gland, which supplies lipids to form the oily layer of the tear film. We found that the lack of CS significantly augmented ocular inflammation induced by ultraviolet irradiation or antigen stimulation ^(23)^.

Certain tissues or organs in our body exhibit a reduced immune surveillance mechanism, the phenomenon known as “immune privilege.” This was first proposed by Sir Peter Medawar (1960 Nobel Laureate in Physiology or Medicine) in 1948, but the precise mechanism of immune privilege has remained elusive for over 70 years. We have identified CS as an endogenous DOCK2 inhibitor. This discovery may provide important clues to elucidate the molecular basis for immune privilege and facilitate its clinical application.

## Atopic Dermatitis and DOCK8

DOCK8 is a Cdc42-specific GEF ^(24)^, and its mutations in humans cause combined immunodeficiency characterized by recurrent viral infection, early-onset malignancy, and atopic dermatitis ^(25)^. IL-31 is currently regarded as one of the most critical pruritogens associated with atopic dermatitis. IL-31 is predominantly produced by helper T cells, but the mechanisms controlling IL-31 production have remained poorly understood. To examine the role of DOCK8 in IL-31 production, we generated AND TCR-transgenic (Tg) mice lacking DOCK8 expression (*Dock8^–/–^* AND-Tg). Surprisingly, these mice spontaneously developed severe dermatitis with scratching behavior and exhibited markedly elevated serum levels of IL-31 ^(26)^. Upon antigen stimulation, helper CD4^+^ T cells from *Dock8^–/–^* AND-Tg mice produced remarkably more IL-31 than those from DOCK8-expressing controls (*Dock8^+/–^* AND-Tg) mice, indicating that DOCK8 negatively regulates IL-31 production.

Next, we compared gene expression profiles between *Dock8^+/–^* CD4^+^ T cells and *Dock8^–/–^* CD4^+^ T cells obtained from AND-Tg mice. Among 856 genes upregulated in the absence of DOCK8, 40 genes encoded transcription factors. Through functional screening, we have identified EPAS1 as a key molecule controlling *Il31* gene expression. Indeed, suppressing *Epas1* expression in *Dock8^–/–^* CD4^+^ T cells markedly inhibited IL-31 production ^(26)^. Moreover, the development of atopic dermatitis was completely abrogated in *Dock8^–/–^* AND-Tg mice when *Epas1* expression was selectively deleted in T cells ^(26)^. EPAS1 is known to mediate hypoxic responses in cooperation with aryl hydrocarbon receptor nuclear translocator (ARNT); however, we found that SP1, but not ARNT, is required for EPAS1-driven IL-31 induction. In contrast, DOCK8 interacts with mammalian ste20 like kinase 1 and inhibits the nuclear translocation of EPAS1. Importantly, this inhibitory effect is mediated by DOCK8 independently of its GEF activity. Thus, we have identified EPAS1 as a therapeutic target for controlling IL-31 production. We are currently trying to develop small-molecule compounds that can inhibit IL-31–associated itch without affecting hypoxic responses.

## Article Information

This article is based on the study that received the Medical Award of The Japan Medical Association in 2024. This is a revised English version of the article originally published in Japanese in the Journal of the Japan Medical Association 2025; 153(10):1103-6 ^(27)^. The original version is available at https://www.med.or.jp/cme/jjma/newmag/15310/15310.html. Only members of the Japan Medical Association are able to access it.

### Conflicts of Interest

None
